# The prognostic value of preoperative serum albumin in patients with bladder urothelial carcinoma undergoing transurethral resection of bladder tumor: A prospective cohort study

**DOI:** 10.1097/MD.0000000000026548

**Published:** 2021-07-09

**Authors:** Chen Shen, Kechong Zhou, Wei Wang, Yue Zhang, Xiaoqiang Liu

**Affiliations:** a Department of Urology, Tianjin Medical University General Hospital, Tianjin, China; b Department of Urology, the Second Hospital of Dalian Medical University, Dalian, Liaoning, China.

**Keywords:** albumin, bladder urothelial carcinoma, non-muscle-invasive bladder cancer, outcome, prognosis

## Abstract

**Background:**

To evaluate whether the preoperative serum albumin level can predict the survival outcome in patients with bladder urothelial carcinoma (BUC) undergoing transurethral resection of bladder tumor (TURBT).

**Methods:**

Four hundred fifty six newly diagnosed patients with BUC who underwent TURBT between January 2014 and December 2017 were retrospectively enrolled. Patients were categorized into low albumin (<40 g/L) and high albumin (≥40 g/L) groups. Survival was estimated using the Kaplan–Meier method and compared using the log-rank test. Univariate and multivariate Cox proportional analyses were used to determine the hazard ratios (HRs) for overall survival (OS). Of patients with available data, 108 (24%) and 348 (76%) patients were classified into the low albumin (<40 g/L) and high albumin (≥40 g/L) groups, respectively.

**Results:**

The results of the Kaplan–Meier analysis and log-rank test showed a significantly worse 5-year OS (*P* = .003) in the low albumin group than in the high albumin group. In the multivariate Cox regression analysis, after adjusting for confounding variables, the preoperative albumin level remained an independent predictor for 5-year OS (HR: 0.434, 95% confidence interval: 0.221–0.852; *P* = .015).

**Conclusion:**

Our study determined that a low preoperative albumin level predicted poor OS in patients with BUC who underwent TURBT. Preoperative serum albumin is an inexpensive and easily available marker that has the potential to be a good prognostic factor for predicting mortality in patients with BUC treated with TURBT.

## 1 Introduction

Bladder urothelial carcinoma (BUC) is the ninth most common malignant disease in humans and the second most common cancer of the genitourinary tract.^[1]^ Transurethral resection of bladder tumor (TURBT) is the standard procedure for the treatment of primary non-muscle-invasive bladder cancer (NMIBC).^[2]^ Currently, the tumor stage and grade are considered to be the major prognostic factors for patients with BUC^[3–6]^; however, these pathologic factors can only be confirmed after surgery. Reliable preoperative prognostic factors have yet to be found for therapeutic decision-making.

Albumin is a major component of human serum proteins. Albumin is synthesized by the liver and is often used to assess the nutritional status of patients. Prior studies have found that the serum albumin levels in patients with high-grade cancers are often low.^[7]^ Some studies have demonstrated that the serum albumin level before surgery or medical treatment is of prognostic value in cancer patients.^[8]^ Accordingly, an increasing number of studies have focused on the prognostic value of preoperative albumin levels in patients with urothelial carcinoma.^[9]^ Some studies have demonstrated that a low preoperative albumin level can be a poor prognostic factor for bladder cancer^[10–12]^; however, others have provided different or contradictory results.^[13,14]^ Consequently, the value of preoperative serum albumin levels as a predictor is still unknown. Further, most of the relevant studies focused on patients undergoing radical cystectomy, and little evidence is available for patients undergoing TURBT. Therefore, this study aimed to evaluate the relationship between the preoperative serum albumin level and outcome in patients with BUC undergoing TURBT.

## 2 Material and methods

### 2.1 Patients

The electronic medical records of patients admitted to the Second Hospital of Dalian University from January, 2014 to December, 2017 were retrospectively reviewed. In total, 456 adult patients were clinically diagnosed with bladder cancer and were included in our database. They were all diagnosed with NMIBC, treated with TURBT, and their post-treatment pathologic specimens were evaluated as BUC. None of the included patients received neoadjuvant chemotherapy or instillation therapy before TURBT. This study was approved by the ethics committee of the Second Hospital of Dalian University. All patients participating in this retrospective study signed informed consent forms.

### 2.2 Data collection

The patient characteristics and preoperative data were obtained from the patient records. Patient demographic data included age, sex, smoking status, Karnofsky score, and American Society of Anesthesiologists (ASA) score. Pathological characteristics included tumor multiplicity, pathological T stage, and grade. Pathologic specimens were evaluated by several pathologists of the pathology department in the Second Hospital of Dalian University. All patients enrolled in this study had undergone routine preoperative laboratory measurements, which included the hemoglobin concentration, neutrophil count, lymphocyte count, platelet count, albumin level, and serum creatinine level. In addition, the creatinine clearance rate was calculated using the Cockcroft–Gault formula, after which the chronic kidney disease (CKD) stage was graded.

### 2.3 Albumin groups

The patients were categorized into low (<40 g/L) and high (≥40 g/L) albumin groups based on the preoperative levels. Of the patients with available preoperative serum albumin data, 108 were in the low albumin group and 348 were in the high albumin group.

### 2.4 Follow-up

The patients were followed up every 3 months for the first 2 years, every 6 months for the third and fourth years, and annually thereafter. Follow-up consisted of cystoscopy, abdominal ultrasonography, chest radiography, and serum chemistry evaluation. If necessary, computed tomography scans and positron emission computed tomography were used.

### 2.5 Statistical analyses

The Statistical Package for the Social Sciences, version 20.0 (SPSS Inc., Chicago, IL, USA) was used for the statistical analyses. The Chi-squared test, Mann–Whitney *U* test, and *t* test were used to compare the differences between the low and high albumin groups. Survival was estimated using the Kaplan–Meier method and compared using the log-rank test. Univariate and multivariate Cox proportional analyses were used to identify the hazard ratios (HRs) for overall survival (OS). A two-tailed *P* value of <0.050 was considered statistically significant for all analyses.

## 3 Results

### 3.1 Patient characteristics

The clinicopathological and laboratory characteristics of the study patients are summarized in Table 1. The patients’ median age was 59.0 years (interquartile range: 38.0–69.0 years). Of all patients, 365/456 (78.8%) were male and 91/456 (19.7%) were female (Table 1). Regarding smoking status, 275 patients had never smoked and 181 patients had a history of smoking. The low albumin group was significantly older than the high albumin group (*P* < .001). There were no significant differences between the groups in terms of sex, smoking status, Karnofsky score, tumor multiplicity, pathological T stage, neutrophil counts, lymphocyte counts, and platelet count (Table 1). However, the high albumin group had a significantly better ASA score, tumor grade, hemoglobin concentrations, and serum albumin levels than the low albumin group, whereas the low albumin group had more patients with a high CKD stage (*P* = .001).

**Table 1:** Characteristics stratified by albumin level in 456 patients.

		Total patients (n = 456) (%)	Low albumin group (n = 108) albumin <40	High albumin group (n = 348) albumin ≥40	*P* value
Clinical characteristic					
Median follow-up	Month (IQR)	59 (38.69)	51 (22.69)	60 (40.70)	.145
Age	Year (IQR)	66 (59.75)	70 (62.78)	65 (58.73)	.001
Gender	Male	365 (78.8)	82 (75.9)	283 (81.3)	.221
	Female	91 (19.7)	26 (24.1)	65 (18.7)	
Smoking status	Smoked in the past	181 (39.7)	48 (44.4)	133 (38.2)	.248
	Never smoked	275 (60.3)	60 (55.6)	215 (61.8)	
Karnofsky score	<90	99 (21.7)	30 (27.8	69 (19.8)	.16
	≥90	357 (78.3)	78 (72.2)	279 (80.2)	
ASA score	1–2 score	366 (80.3)	75 (69.4)	291 (83.6)	.001
	3–5 score	90 (19.7)	33 (30.6)	57 (16.4)	
Pathological characteristic					
Tumor multiplicity	Unifocal	287 (62.9)	69 (63.9)	218 (62.6)	.815
	Multifocal	169 (37.1)	39 (36.1)	130 (37.4)	
Pathological T stage	Ta	103 (22.6)	18 (16.7)	85 (24.4)	.092
	T1	353 (77.4)	90 (83.3)	263 (75.6)	
Grade	Low	213 (46.7)	35 (32.4)	178 (51.1)	.001
	High	243 (53.3)	73 (67.6)	170 (48.9)	
Laboratory					
Hemoglobin	g/L	140.99 ± 17.66	129.23 ± 19.04	144.64 ± 15.52	<.001
Neutrophil count	G/L	3.95 ± 1.52	4.09 ± 1.90	3.9 ± 1.39	.913
Lymphocyte count	G/L	1.98 ± 0.69	1.92 ± 0.78	2 ± 0.66	.064
Platelet count	G/L	213.98 ± 58.95	205.4 ± 74.75	216.87 ± 52.43	.187
Albumin	g/L	42.78 ± 4.31	36.91 ± 3.39	44.48 ± 2.79	
CKD	1–2 grade	375 (82.2)	77 (71.3)	298 (85.6)	.001
	3–5 grade	77 (17.8)	30 (27.8)	47 (13.5)	
	Missing	4 (0.9)	1 (0.01)	3 (0.01)	

CKD = chronic kidney disease, IQR = interquartile range.

### 3.2 Univariate analysis

**Figure 1:**
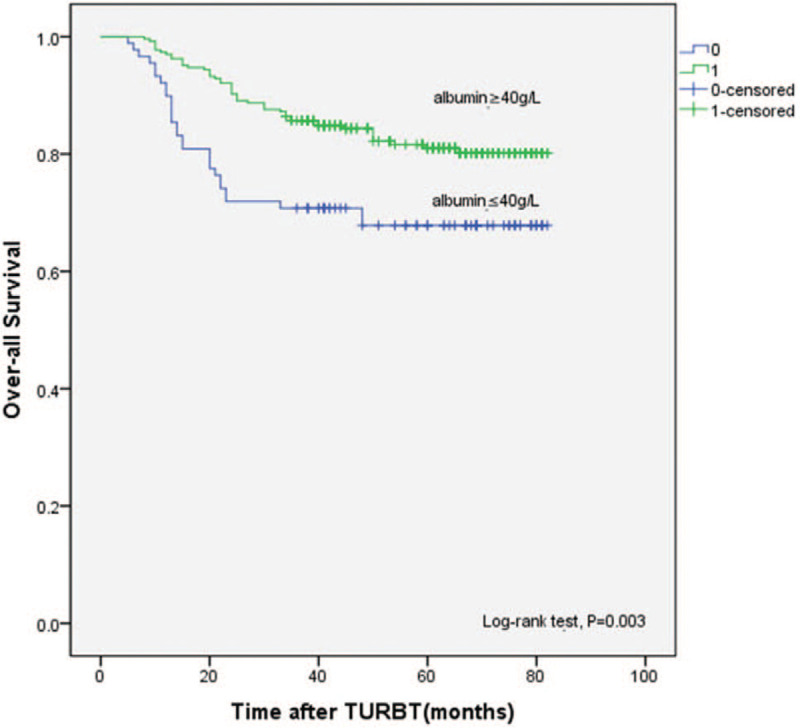
Comparison of OS according to preoperative serum albumin in 456 patients. OS = overall survival.

The median follow-up period was 59 months (interquartile range: 38–69 months). During the follow-up, 76 patients died; the mortality rates in the low and high albumin groups were 25.9% (28/108) and 13.8% (48/348), respectively. The results of the Kaplan–Meier analysis and log-rank test showed a significantly worse 5-year OS (*P* = .003) in the low albumin group than in the high albumin group (Fig. 1).

The univariate Cox proportional analysis results are shown in Table 2. There was a statistically significant relationship between the preoperative serum albumin level and OS. Among the demographic and clinicopathological variables, age, Karnofsky score, and ASA score were significantly associated with OS. Among the laboratory variables, hemoglobin level, lymphocyte count, albumin level, and CKD stage were significantly associated with OS.

**Table 2:** Univariate and multivariate analysis regarding OS in 456 patients.

		Univariate analysis		Multivariate analysis	
		HR (95% CI)	*P* value	HR (95% CI)	*P* value
Clinical characteristic					
Age	<65	1		1	
	≥65	1.26 (1.208, 1.306)	<.001	1.216 (1.124 ,1.317)	<.001
Gender	Female	1		1	
	Male	0.933 (0.538, 1.620)	.806	0.566 (0.255, 1.258)	.163
Karnofsky score	≥90	1		1	
	<90	3.057 (1.429, 6.536)	.004	3.511 (1.483, 8.314)	.004
ASA score	1–2 score	1		1	
	3–5 score	34.664 (19.612, 61.267)	<.001	2.482 (0.255, 1.258)	.163
Pathological characteristic					
Tumor multiplicity	Unifocal	1		1	
	Multifocal	1.116 (0.706, 1.763)	.639	1.136 (0.602, 2.141)	.694
Pathological T stage	Ta	1		1	
	T1	0.920 (0.541, 1.562)	.757	1.369 (0.414, 4.523)	.606
Grade	Low	1		1	
	High	1.508 (0.949, 2.396)	.082	1.081 (0.522, 2.242)	.833
Laboratory					
Hemoglobin		0.988 (0.977, 0.999)	.027	0.988 (0.964, 1.011)	.304
Neutrophil count		1.040 (0.903, 1.197)	.588	1.000 (0.864, 1.158)	.997
Lymphocyte count		0.651 (0.451, 0.938)	.021	0.804 (0.472, 1.370)	.423
Platelet count		0.997 (0.992, 1.002)	.204	0.998 (0.991, 1.004)	.474
Albumin	Low	1		1	
	High	0.501 (0.314, 0.799)	.004	0.434 (0.221, 0.852)	.015
CKD	1–2 grade	1		1	
	3–5 grade	7.751 (4.876, 12.321)	<.001	1.009 (0.509, 2.001)	.978

CI = confidence interval, CKD = chronic kidney disease, HR = hazard ratio.

### 3.3 Multivariate analysis

All potential valuable variables were included in the multivariate survival analysis to determine whether the preoperative serum albumin level was an independent prognostic factor for BUC (Table 2). Table 2 shows that the preoperative serum albumin level (HR: 0.434, 95% confidence interval [CI]: 0.221–0.852; *P* = .015), age (HR: 1.216, 95% CI: 1.124–1.317; *P* < .001), and Karnofsky score (HR: 3.511, 95% CI: 1.483–8.314; *P* = .004) remained independent prognostic factors for OS in the multivariate analysis.

## 4 Comment

BUC is a common malignancy with a high recurrence rate.^[1]^ TURBT is the standard of care for treating patients with primary NMIBC.^[2]^ In recent years, several novel preoperative prognostic indicators for outcomes of BUC have been investigated; however, most of them have limitations in their clinical applicability, particularly in terms of the high cost associated with their detection methods.^[15,16]^ As such, this study focused on the prognostic value of preoperative serum albumin levels, which are readily available and inexpensive to obtain.

Hypoalbuminemia is believed to be a negative prognostic factor for patients undergoing various surgical procedures.^[17]^ Prior studies found that low preoperative serum albumin levels can predict poor survival in patients with BUC^[11,12,18]^; however, to the best of our knowledge, our study is the first to investigate the relationship between the preoperative albumin level and OS in Chinese patients with BUC undergoing TURBT.

In the present study, the preoperative albumin level, age, and Karnofsky score remained independently associated with OS in the multivariate analysis. Conversely, tumor multiplicity, pathological T stage, and tumor grade were no longer statistically significant prognostic factors in the multivariate analysis. The predictive efficacies of age have been demonstrated in many previous reports,^[19–21]^ and the Karnofsky score has also been shown to be associated with patient age, underlying disease, performance status, and nutrition status, and may also be indicative of patient outcome.^[14]^

Albumin is a product released by the liver, and the serum albumin level has been shown to be lower in patients with various cancers.^[7,8]^ As albumin is a commonly used marker for assessing nutrition status, a low level of albumin can indicate malnutrition status. Many studies have investigated the relationship between the preoperative albumin level and complications during the perioperative period. Hollenbeck and colleagues analyzed National Surgical Quality Improvement Program data from 1991 to 2002. The researchers found that patients who underwent radical cystectomy and TURBT and also had a low preoperative albumin level suffered more complications and had a high rate of mortality.^[22,23]^ Johnson et al performed a retrospective review of the National Surgical Quality Improvement Program (2005–2012) data and discovered that a low albumin level was a powerful predictor for postoperative complications. This result remained significant when the preoperative albumin level was evaluated as a continuous variable.^[24]^ Using data from an electronic hospital-based surgical morbidity database, Garg et al conducted a study on radical cystectomies performed in patients with BUC and found that the risk of complications and mortality increased when the serum albumin level decreased.^[25]^ In all of these studies, malnutrition was considered the primary reason for the high rate of postoperative complications. Since the defense mechanisms are weakened in patients with malnutrition, complications and rapid progression of the cancer are more common. Therefore, a low albumin level is often associated with poor survival. We consider that low preoperative albumin levels not only represent poor nutritional status but also weak self-defense mechanisms, which lead to poor short-term prognosis and long-term outcome.

Aside from being a nutritional status marker, albumin might be related to the inflammatory response.^[26]^ Many studies have investigated the influence of the inflammatory response on bladder cancer progression and found that some inflammatory biomarkers, such as C-reactive protein, play important roles in the mortality of cancer patients.^[27–29]^ Further, some studies have found that cytokines released by tumors, such as interleukin (IL)-6, can decrease the production of albumin by blocking the production passageway. Increased levels of tumor necrosis factor-α can selectively inhibit the genetic expression of albumin, which can lead to a decrease in the serum albumin level.^[30]^ There are 3 important ways in which these inflammatory factors are expressed. First, growth and invasion of the cancer tissue can activate the inflammatory response in surrounding tissues and stimulate human immune cells to release inflammatory factors. Second, cancer cells can synthesize inflammatory cytokines, such as IL-1, IL-6, IL-8, tumor necrosis factor-α, and vascular endothelial growth factor, independently. Third, an insufficient blood supply, necrosis, or hypoxia of the tumor may cause an increase in cytokines. Therefore, these 3 mechanisms can reduce the production of albumin and explain why low preoperative albumin levels can be regarded as a marker of systemic inflammation and a poor prognostic factor for the survival of cancer patients.

Considering the important role of preoperative serum albumin in assessing nutritional status and the inflammatory response, we consider that the preoperative albumin level also associates with the progression and metastasis of BUC in patients who undergo TURBT. Patients with low preoperative albumin levels (i.e., <40 g/L) should have the more intensive treatment and closely monitored follow-up care.

Our study has some limitations. First, this was a single-center retrospective study with small sample size. Second, there may be recall bias in our study because telephone calls were used as the primary method for long-term prognosis follow-up. Third, different surgical skills among surgeons may have affected the patients’ prognosis. Further multicenter studies with large patient cohorts are needed to confirm our findings.

## 5 Conclusions

In conclusion, we consider that the preoperative serum albumin level can be a good prognostic factor for patients with bladder cancer because it plays an important role in evaluating the nutritional status and the systemic inflammatory response. Our study determined that a poor preoperative albumin level can predict poor OS in bladder cancer patients undergoing TURBT. Future prospective multicenter studies with a larger sample size are needed to verify the prognostic value of the preoperative albumin level for patients with bladder cancer treated with surgery.

## Acknowledgments

We sincerely thank Li Liu (Tianjin Medical University General Hospital) and Lei Huang (Tianjin Medical University General Hospital) for the help and support of the study.

## Author contributions

**Administrative support:** Chen Shen and Kechong Zhou.

**Collection and assembly of data:** Chen Shen, Kechong Zhou, Wei Wang, Yue Zhang.

**Conception and design:** Chen Shen and Kechong Zhou.

**Conceptualization:** Chen Shen.

**Data analysis and interpretation:** Chen Shen, Kechong Zhou, Wei Wang, Yue Zhang, Xiaoqiang Liu.

**Data curation:** Chen Shen.

**Final approval of manuscript:** All authors.

**Formal analysis:** Chen Shen.

**Funding acquisition:** Xiaoqiang Liu.

**Investigation:** Kechong Zhou.

**Manuscript writing:** All authors.

**Methodology:** Kechong Zhou.

**Project administration:** Kechong Zhou.

**Provision of study materials or patients:** Chen Shen and Wei Wang.

**Resources:** Wei Wang.

**Software:** Wei Wang.

**Supervision:** Yue Zhang.

**Validation:** Yue Zhang.

**Visualization:** Yue Zhang.

**Writing – original draft:** Chen Shen, Kechong Zhou, Wei Wang, Xiaoqiang Liu.

**Writing – review & editing:** Chen Shen, Kechong Zhou, Xiaoqiang Liu.
